# Sign language delays in deaf 3- to 5-year-olds with deaf parents

**DOI:** 10.1093/deafed/enad059

**Published:** 2023-12-11

**Authors:** Donna A Morere, Thomas E Allen, Maura Jaeger, Dana Winthrop

**Affiliations:** Department of Psychology, Gallaudet University, Washington, D.C., United States; Science of Learning Center on Visual Language and Visual Learning, Gallaudet University, Washington, D.C., United States; Science of Learning Center on Visual Language and Visual Learning, Gallaudet University, Washington, D.C., United States; Educational Neuroscience Program, Gallaudet University, Washington, D.C., United States; Center for Language Science, Penn State University, State College, PA, United States; Center for Language Science, Penn State University, State College, PA, United States

## Abstract

Research has demonstrated that deaf children of deaf signing parents (DOD) are afforded developmental advantages. This can be misconstrued as indicating that no DOD children exhibit early language delays (ELDs) because of their early access to a visual language. Little research has studied this presumption. In this study, we examine 174 ratings of DOD 3- to 5-year-old children, for whom signing in the home was indicated, using archival data from the online database of the Visual Communication and Sign Language Checklist. Our goals were to (1) examine the incidence of ELDs in a cohort of DOD children; (2) compare alternative scaling strategies for identifying ELD children; (3) explore patterns among behavioral ratings with a view toward developing a greater understanding of the types of language behaviors that may lie at the root of language delays; and (4) suggest recommendations for parents and professionals working with language-delayed DOD children. The results indicated that a significant number of ratings suggested ELDs, with a subset significantly delayed. These children likely require further evaluation. Among the less delayed group, ASL skills, rather than communication or cognition, were seen as the major concern, suggesting that even DOD children may require support developing linguistically accurate ASL. Overall, these findings support the need for early and ongoing evaluation of visual language skills in young DOD children.

Early language delays (ELDs) may be persistent or transient; those whose delays continue through age 5 are more likely to represent a developmental language disorder (DLD, also called a specific language impairment or primary language impairment). Many children with ELDs achieve adequate language skills by age 5. Thus, while 11%–18% of children may demonstrate ELDs, only 3%–7% of all children approaching kindergarten age are identified as having a DLD (Chilosi et al., 2021; Laasonen et al., 2018; Sansavini et al., 2021). The DLDs are associated with ongoing limitations in language, literacy and academic functioning, and psychosocial functioning, as well as limitations in cognitive functions such as serial short-term memory (linguistic and nonlinguistic), working memory, and processing speed (Laasonen et al., 2018; Lahti-Nuuttila, Laasonen et al., 2021; Lahti-Nuuttila, Service et al., 2021; Matte-Landry et al., 2020). While these relationships can be causative, as in the case of limitations in social relationships and reduced self-esteem due to communication deficits, others may reflect broader conditions, such as cognitive impairments for which DLD may be a symptom rather than the primary condition (Laasonen et al., 2018). Laasonen and colleagues noted that DLD could be the result of deficits in underlying cognitive skills. Indeed, Lahti-Nuuttila and colleagues found that nonverbal sequential short-term memory performance was predictive of language development outcomes in children with DLD, but not those with typically developing language. Regardless of the causative or secondary nature of ELD/DLD, early identification can alert clinicians to the potential presence of other conditions requiring intervention.

Early identification is critical to facilitate the implementation of interventions that may ameliorate the effects of both ELD and DLD and other potential conditions, and screenings beginning between ages two and three are recommended and diagnosis is anticipated by about age 4 (Sansavini et al., 2021). In contrast, deaf and hard-of-hearing (DHH) children may have ongoing language issues, but the diagnosis of a DLD may be delayed or absent. While language delays, both spoken and signed, are common in children who are born DHH, for those with hearing parents, this is often attributed to delayed, inadequate, or inaccessible language modeling resulting in language deprivation (Glickman et al., 2020; Hall, 2017; Hall et al., 2019; Howerton-Fox & Falk, 2019; Marshall & Morgan, 2016). However, children, both DHH and hearing, may have delays in language development for a variety of reasons in addition to inadequate accessible exposure, including intellectual disabilities (IDs), autism spectrum disorder (ASD), or other neurodevelopmental conditions; factors such as low parental education; visual impairment or visuospatial processing disabilities; and DLD (Buschmann et al., 2008; Marshall et al., 2020; Sunderajan & Kanhere, 2019). While visual impairment and visuospatial processing deficits would not typically be expected to affect language development, due to the visuospatial nature of American Sign Language (ASL), this can affect language development in signing deaf children (Morere, 2010; Quinto-Pozos et al., 2013). Similarly, while children may “overhear” language modeling regardless of where their attention is focused, DHH children must visually attend to signed communication in order to benefit from modeling of ASL. Thus, ASL development of deaf children with atypical attention or gaze, such as those with attention-deficit hyperactivity disorder (ADHD) or ASD, may be hindered, although these factors in isolation would be expected to produce delays rather than serious impairment (Morere, 2010; Quinto-Pozos et al., 2017).

Despite the complexity of identification of ELD in signing deaf children, recent research has identified language disorders in DHH signers, particularly those who would typically be expected to have adequate language development, such as those with deaf signing parents (Henner et al., 2018; Herman et al., 2014; Marshall & Morgan, 2016; Mason et al., 2010; Morere, 2003; Morgan et al., 2007; Quinto-Pozos et al., 2011, 2017; Shield et al., 2017; Woll & Morgan, 2012). Prevalence of developmental disabilities (including deafness) in children ages 3–7 in the USA is estimated to be 17.8% (Micai et al., 2020). In contrast, it is estimated that 35%–50% of deaf children have an additional disability (Mitchell & Karchmer, 2006). While many causes of deafness may be associated with additional conditions, the majority of such etiologies (e.g., cytomegalovirus or meningitis) are associated with deaf children from hearing families. Fewer risks are associated with children from deaf families (with presumed genetic etiologies), whose risks of language delays would be expected to be more similar to those in the general population reported by Micai and colleagues.

Regardless of the source, language delays can have long-term consequences for DHH children, affecting social and academic outcomes (Buschmann et al., 2008; Kaiser & Roberts, 2011; McLaughlin, 2011). As early intervention can ameliorate, although not necessarily resolve, language delays, early identification, and intervention are critical; thus, the American Academy of Pediatrics recommends regular screening of infants and toddlers for ELDs (Mackrides & Ryherd, 2011). Indeed, ELDs caused by environmental factors may be resolved through early intervention; however, despite improvements resulting from early intervention, DLD will likely have enduring effects on functioning. In their systematic review of the literature, Micai et al. (2020) found that screening of language development by age 3 was one of the key factors in the early identification of neurodevelopmental disorders such as ASD, ADHD, and learning disabilities. Buschmann et al. (2008) found that identification of ELD at age 2 was a sensitive marker for a range of developmental issues, enabling early interventions to address not only language delays but also any other disabilities that may be present. Related conditions cited by Micai and colleagues and Buschmann et al. include ASD, ADHD, learning disabilities, impairment of nonverbal cognitive abilities, and general cognitive impairment. Thus, the early identification of language delays in DHH children is critical, and accurate measures of early ASL development are needed in order to identify ELD/DLD in signing DHH children.

While multiple early screening tests and rating scales targeting children ages 5 years and younger are available to monitor the language development of hearing children (Scott et al., 2016), few have been developed and standardized for use with DHH children, particularly those whose language exposure is primarily via ASL. While some measures of natural signed languages have recently been developed to evaluate the skills of older children and adults (Enns, et al., 2016; Enns & Herman, 2011; Enns & McQuarrie, 2021; Henner et al., 2018; Mann & Prinz, 2006; Paludneviciene et al., 2012), little attention has been given to early signed language development. Although the ASL-CDI 2.0, a parent report, does provide information about ASL vocabulary development via a large database of deaf children of deaf signing parents (DOD) through 73 months (Caselli et al., 2020), measures reflecting the range of early ASL and visual communication skills would provide more comprehensive information.

A lack of early measures that would allow for the monitoring of appropriate development of ASL is a major obstacle to early identification and intervention for DHH children experiencing language delays as well as the identification of potential sources of these delays. One measure that has been developed is the Visual Communication and Sign Language Checklist (VCSL; Simms et al., 2013). Since its publication, the VCSL has been widely used and was recently reported to be the most popular measure of ASL development in young deaf children (Secora et al., 2022). Secora et al. surveyed professionals, including speech language pathologists, teachers of the deaf, and ASL specialists, working with young DHH children. They reported that more than 80% of the respondents relied on observation or elicited language samples, while other measures, such as the Kendall Conversational Proficiency Levels, Social Communication Skills-Pragmatics Checklist, and Language Development Scale, followed the VCSL in frequency of use. The VCSL, and its online version (VCSL:O, Allen & Morere, 2022) are described more fully below. The current study presents an analysis of VCSL:O data.

While ELD are considered typical of DHH children with hearing parents (DOH) due to both language modeling and etiological issues, deaf children with deaf parents (DOD) are generally expected to demonstrate sign language development and acquisition of language milestones in a manner consistent with hearing children’s development of spoken language (Lillo-Martin & Henner, 2021; Marshall & Morgan, 2016; Petitto & Marentett, 1991; Pichler, 2002). For example, Lillo-Martin and Henner (2021, p. 397) state that “when learners have early access to fluent signers, their development of a sign language progresses along the same timeline as expected given previous research on the development of a variety of spoken languages.” Despite this expectation, DOD children are also vulnerable to a range of causes of ELD, including visuospatial issues, neurodevelopmental disabilities, and DLD. Furthermore, while culturally deaf parents, particularly those from multigenerational deaf families, may be fluent in ASL, many deaf parents may themselves have been DOH and had limited parental sign models, attended educational programs that used some form of English-based signing or simultaneous communication, or been late signers; thus, having developed less linguistically accurate ASL skills, reflected in linguistic and grammatical features (e.g., reduplication, use of classifiers, non-manual markers) typically evaluated using measures of ASL, including the VCSL (Singleton & Meier, 2021; Singleton & Newport, 2004; Sümer & Özyürek, 2022). This could be compounded if the child has limited access to additional, more linguistically accurate, models of ASL. Finally, considering the incidence of disabilities among the deaf population, some deaf parents may have disabilities that affect their language modeling for their deaf child or the child may have a disability that limits their ability to take advantage of the modeling. As with the general population, there are multiple potential sources of ELD and DLD among DOD children. Thus, it is critical that the ASL skills of both DOH and DOD children be monitored. Even so, DLD in DOD children have only recently been investigated, primarily though case studies (Marshall & Morgan, 2016; Morere, 2003; Quinto-Pozos et al., 2017; Quinto-Pozos & Cooley, 2020).

Studies of ASL-based DLD have reported linguistic structures that appear to be affected in such conditions, including syntax, morphology, use of classifiers and sign space, and narrative skills (Quinto-Pozos & Cooley, 2020). Furthermore, some conditions may produce specific sets of atypicalities in ASL performance. For example, deaf children with ASD have been found to reverse the orientation of their palm and the direction of movement (e.g., producing *Tuesday* rather than *toilet* or *paper* rather than *clean*) when signing; these types of errors are highly unusual for typically developing deaf children (Shield et al., 2021; Shield & Meier, 2014). Shield and colleagues further cited research indicating that children with ASD were also reported to reverse palm orientation during fingerspelling despite fewer errors in handshape production. This is hypothesized to be related to issues with perspective-taking rather than issues with production or motor control. Shield et al. (2017) also found increased reduplication among deaf children with ASD, a behavior that can alter the meaning (e.g., noun–verb status) of the sign. This latter may be a product of perseveration, a behavior common in individuals with ASD.

While many of these factors may also be seen in DHH children with ELD due to reasons other than a DLD in isolation, their presence in early ASL screenings may aid in discriminating among DHH children whose ELD are secondary to environmental influences (e.g., inadequate modeling), neurodevelopmental conditions other than DLD (e.g., ASD or ID), and DLD. For example, DOD children whose parents were late signers have been reported to use fewer classifiers despite demonstrating more advanced ASL skills than their late signing parents (Sümer & Özyürek, 2022). In contrast, children with ELD secondary to ASD may demonstrate limitations in the development of socioemotional aspects of communication as well as specific atypicalities of signing frequently seen with DHH children with ASD (Shield et al., 2021; Shield & Meier, 2014).

The current study investigates deidentified data from a large cohort of online ratings using the VCSL:O with a focus on DOD children ages 3–5 using ASL in the home whose ratings indicate at least a 2-year delay in ASL skill development. The goals were to

(1) Examine the incidence of ELD among a national sample of DOD children ages 3–5 from homes reporting the use of ASL.
(2) Examine and compare alternative diagnostic scaling strategies for identifying ELD children, including assessments of the severity of noted delays.
(3) Explore patterns within VCSL ratings among children identified as having ELD that might pinpoint aspects of early language development that are particularly problematic for such children and might suggest whether the ELD seen represented environmental factors, other neurodevelopmental conditions, or DLD.
(4) Suggest recommendations for parents and professionals working with these children.

## Methods

### Instrumentation

#### The VCSL: Online

The VCSL is a nationally normed checklist targeted at DHH children ages birth to 5 years old, originally developed as a paper-and-pencil rating scale and later converted to an online version (VCSL:O) using the identical item content of the original, but adding video clips of target item behaviors, adaptive testing, automatic scoring, and other advantages. The 114-item scale is designed to be used by experienced, trained raters who evaluate the child based on standard exemplars of the target language behaviors ordered by developmental level and grouped by age. The raters, typically teachers of the deaf, early interventionists, or other early educational professionals who work directly with the children, are expected to know the child personally and thus be familiar with the child’s language functioning. The children being rated are not asked to demonstrate the relevant skills; rather, the raters’ prior knowledge of their functioning is used to rate the individual child’s skills on each VCSL item. In addition to limiting most access to raters trained on the VCSL, within the VCSL:O, there are video clips of age-appropriate children demonstrating the target skills for each item to enhance scoring accuracy. The items are grouped into age-based sets divided into birth to 1 year (Set 1), 1–2 years (Set 2), 2–3 years (Set 3), 3–4 years (Set 4), and 4–5 years 11 months (Set 5; Simms et al., 2013). Simms and colleagues indicated that items were placed within each year set so that a typically developing child would be expected to master all items within the age set reflecting their chronological age. The items were sequenced such that earlier items within a set were typically mastered at an earlier age (e.g., for Set 2, earlier items might be mastered by 75% of the sample by 2 years, 5 months, while later items were mastered by this proportion of the sample by 2 years 8 months).

Children are rated on each behavior using a four-point scale of 1—not yet emerging (NYE); 2—emerging; 3—inconsistent use; and 4—mastered. The published standard scoring protocol of the VCSL focuses on the NYE and mastered ratings, which are used to generate estimates of basal and ceiling levels (see Simms et al., 2013). More specifically, a child’s basal score was determined by the item that required the greatest level of skill from among a sequence of 10 consecutive items rated as mastered. A ceiling level was determined by the item beyond which 10 items in sequence were rated as NYE. A more recent, statistically derived, scaled score has been developed using a partial-credit Rasch analysis (Masters, 1982; Rasch, 1960) that uses all the rating data (including the emerging and inconsistent use ratings) to provide more detailed information about the child’s skills. The resulting Rasch scaled score has a range from 0 to 100 (Allen & Morere, 2022). In addition to including all four item rating categories, Rasch scoring statistically identifies item difficulty and is not affected by the majority of issues with the published item sequencing within the VCSL. One area that this does not address is the impact of a premature ceiling that could result from a series of harder items preceding those that required lower skill levels. Thus, even with the Rasch scoring, it is possible that some ratings underestimate a child’s skills. This method does, however, allow for scoring of ratings for which a basal or ceiling could not be determined and provides standard scores that allow for comparisons across items and individual ratings.

#### Item categories

Items were categorized as reflecting a number of underlying functions based on the task demands. Some items involved multiple functions. For example, items such as combining nouns and verbs or using three to four sign sequences could reflect both expressive language and ASL; however, they could simply represent expressive language without the grammatical features of ASL. Thus, they were classified as expressive language rather than ASL. Responding to a question could conceivably involve ASL (if that was how the question was presented or answered), both receptive and expressive language, and the cognitive demands of analyzing the question and formulating a response. The latter was considered the most fundamental task demand, as a child could understand the question (whether ASL or other signing) but not perform the required analyses to formulate a response. Similarly, while they might have the expressive skills to respond, if they were not able to perform the underlying cognitive analysis, the response could be poorly developed or unrelated to the question, resulting in a lower rating. The category considered to reflect the key function for that item was identified as the primary category and used for the analyses. The categories included expressive language (e.g., number of signs used or multisign utterances), receptive language (e.g., understanding signed or fingerspelled words), cognitive (e.g., formulating stories and understanding opposites, numbers, and timelines), ASL (e.g., using various classifiers, nonmanual markers, and handshapes and using ASL sequencing or grammatical structures such as verb modification and conditionals), social/emotional (e.g., smiling and laughing, enjoying physical contact), attention & gaze (e.g., following eye gaze, visually focusing on speaker or objects), prelinguistic/visual–gestural communication (e.g., hand babbling, using gestures for communication), and pragmatics (e.g., understanding turn taking). Prelinguistic, attention and gaze, and social–emotional behaviors primarily occurred within the birth to 1-year item set. Prelinguistic behaviors included hand play, copying others’ movements, hand and finger babbling, and using gestures to communicate, behaviors that typically precede expressive signing. Attention and gaze items include looking attending to, fixating on, and tracking faces, signs, and movements; looking in the direction of pointing; and making eye contact. These activities are prerequisites for receptive signing. Social–emotional activities included distinguishing facial expressions, expressing emotions, participating in communicative play, and enjoying social interactions. These activities are important for understanding and expressing connotations and social meaning in addition to literal understanding of language.

Language functions were divided into expressive language, receptive language, and ASL. The classification of ASL was restricted to items for which a rating of mastery would require core ASL features such as grammatically correct sequencing, use of space and classifiers, verb modifications, or nonmanual markers. The receptive and expressive language labels were used when the tasks did not require use or understanding of ASL characteristics such as grammar. For example, an expressive skill might be demonstrated when a child uses one or more signs to ask for help using manually coded English or attempted ASL without using grammatically correct ASL features. Also, one and two sign utterances may occur without any underlying ASL skills. Indeed, a child can tell a story using visual–gestural communication and signs that are devoid of ASL grammar. Similarly, a child might respond to simple signed questions or commands regardless of whether the information is signed in linguistically accurate ASL or another form of signing or if they understand the grammar of correctly signed ASL. The category of ASL was primarily attributed to expressive behaviors that demonstrate use of either ASL grammatical structures (e.g., reduplication, use of classifiers, ASL sign sequences such as *doggie-where*) or key characteristics of ASL (e.g., use of nonmanual markers, more advanced handshapes, lexicalization, body shift and eye gaze).

The category of cognitive functions is a very broad category that includes numeracy; understanding of concepts such as opposites and similarities, parts, time, and seasons; sequencing or categorizing of items based on characteristics such as color, size, shape; use of cognitive search strategies (e.g., naming items given a category such as animals or foods); and understanding underlying meaning and formulating thoughts to be expressed (e.g., understanding and responding to questions and formulating stories). Often cognitive functions are combined with receptive and expressive language; however, when that occurs, the underlying cognitive skills may be the key, as children with limited language skills may understand others or find ways of conveying the concept they have formulated despite limited or inadequate receptive or expressive language.

### Sample characteristics

One advantage of the online version of the VCSL is that, based on permission granted by the children’s parents at the time of the rating, the resulting item ratings, the basal and ceiling levels, and each child’s background characteristics are retained in a database intended for analysis. The current study employed data on 1,003 independent VCSL evaluations extracted from the online dataset on July 6, 2022. These constituted the entire set of data on children whose parents had granted permission for their ratings to be retained for subsequent research. The ratings were conducted between April 2016 and July 6, 2022. Rasch scaled scores for each rating, using a raw-to-scaled score conversion table created and published in 2022 (Allen & Morere, 2022), were added to the dataset.

In the current paper, we were interested in looking at language delay among the subset of the total dataset comprised of deaf children with one or both parents reported to be deaf and using ASL in the home in the 3- to 5-year-old age range. Children younger than three were excluded as they were too young to demonstrate a 2-year or greater language delay. As can be seen in Table 1, this reduced the total number of ratings to 174, including 73 3-year-olds, 66 4-year-olds, and 35 5-year-olds. As can be seen from the table, while the DOD sample was similar in many ways to the full sample (e.g., gender, age, and congenital status), the DOD sample indicated that ASL was the primary language in the home for all respondents and few of the DOD children were reported to have cochlear implants (CIs). While further information about the parents in the sample would have been valuable, due to the archival nature of the dataset, no further information was available.

**Table 1:** Demographic characteristics of full VCSL sample and relevant analysis subgroups.

**Full sample, *N =* 1,003**			**DOD/ASL, *N =* 259**		**Ages 3–5, *N =* 174**		**Basal delay, *N =* 50**		**Rasch delay, *N =* 33**	
Age in years	*N*	%	*N*	%	*N*	%	*N*	% w/in delay (% w/in age)	*N*	% w/in delay (% w/in age)
0	59	5.9	8	3.1	–	–	–	–	–	–
1	129	12.9	20	7.7	–	–	–	–	–	–
2	174	17.3	42	16.2	–	–	–	–	–	–
3	235	23.4	73	28.2	73	42.0	12	24.0 (16.4)	12	36.4 (16.4)
4	203	20.2	66	25.5	66	37.9	23	46.0 (34.8)	15	45.5 (22.7)
5	146	14.6	35	13.5	35	20.1	15	30.0 (42.8)	6	18.2 (17.1)
6	38	3.8	10	3.9	–	–	–	–	–	–
7	12	1.2	5	1.9	–	–	–	–	–	–
Information not available	7	0.7	–	–	–	–	–	–	–	–
Languages in the home										
All or most ASL—deaf parents	259	25.8	259	100	174	100	50	100	33	100
All or most ASL—both hearing parents	95	9.5	–	–	–	–	–	–	–	–
Equal ASL and spoken English	193	19.2	–	–	–	–	–	–	–	–
All or most spoken English	384	38.3	–	–	–	–	–	–	–	–
Information not available	72	7.2	–	–	–	–	–	–	–	–
Gender										
Female	505	50.3	132	51.0	93	53.4	25	50.0	15	45.5
Male	498	49.7	127	49.0	81	46.6	25	50.0	18	54.5
Born deaf										
No	296	29.5	59	22.8	46	26.4	14	28.0	14	42.4
Yes	707	70.5	200	77.2	128	73.6	36	72.0	19	57.6
Cochlear implant										
No	798	79.6	255	98.5	172	98.9	49	98.0	32	97.0
Yes	205	20.4	4	1.5	2	1.1	1	2.0	1	3.0
Hearing identification of mother (and father)^a^										
Deaf	264	26.3	229	88.4	157 (137)	90.2 (78.7)	45	90.0	31	93.9
Hearing	691	68.9	15	5.8	9 (12)	5.2 (6.9)	2	4.0	—	—
Information not available	48	4.8	15	5.8	8 (25)	4.6 (14.3)	3	6.0	2	6.1

^a^Counts and percentages for father’s hearing identification are shown for the target sample of 174 ratings of DOD 3- to 5-year olds in signing families only. Among the ratings with reported hearing identifications provided for both parents (*N =* 147), a total of 129 ratings were provided (87.8%) for participants for whom both parents were deaf.

### Identification of ELD ratings

The two aforementioned scoring systems (the basal/ceiling system based on consistent ratings of “mastered” to determine basal levels and consistent ratings of “NYE” to determine ceiling levels and the Rasch-based scaled scores providing a continuous equal-interval scale of mastery) were evaluated to identify separate indicators of language delay. The “basal age” was determined by the ordinal position among the 114 behavioral statements that indicated the highest point at which 10 ratings in a row were given a rating of “mastered.” According to Simms et al. (2013), each item position (from 1 to 114) had associated with it a “basal range” that identified the expected ages at which 25%, 50%, and 75% of participants would achieve mastery. Simms et al. (2013) indicated that items were expected to be mastered within the age range of the item set (e.g., all items within Set 1 would be expected to be mastered by age 12 months). Furthermore, the differences between the 50% and 75% mastery were typically only 1–2 months, with the 75% level being achieved by the eighth month of the year for all items beyond Set 1. Thus, we calculated a “basal difference” score that represented the difference between the child’s actual age (in months) and the basal age (in months) associated with an expected 75% level of mastery. We determined that evaluations of children whose chronological age was 24 months (or more) greater than their calculated basal age were indicative of a 2-year language delay.

Using the Rasch scores to determine language delay involved a different strategy. Since all evaluations were assigned a Rasch scaled score ranging from 0 to 100, indicating the level of skill, we calculated (from the original sample used to create the Rasch scores) median Rasch scores separately for 3-, 4-, and 5-year-olds (Allen & Morere, 2022). In addition, means and medians for the full current sample (hearing as well as deaf parents downloaded July 6, 2022) and the current DOD sample were derived. These means and medians are reported below in Table 3. Note that as the sample from Allen and Morere (2022) is a subset of the current full sample, which includes the original ratings as well as the ratings completed between the initial download and July 6, 2022, the means and medians are quite similar for these two groups, while they are at least 6–8 points higher for the current DOD sample. As they represented the normative data for the Rasch scores, the medians from the original Rasch analysis were used for the comparison group for the following analyses. Thus, we then calculated, separately within each age group, the differences between the derived scaled score from each rating and the median level of rating of children who were the same age from the original scaling. Positive deviation scores indicated that the rated child scored above the median for their age group; negative deviation scores indicated that the rated child scored below the age-based median. Upon examining the distributions of scaled scores within each age group, we noted a distinct negative skew. It was evident that there were evaluations in the dataset representing two levels of delay: those whose scores deviated from the age-based medians by 4 or more points (but less than 10), and those whose scores deviated by 10 or more points. These groups were defined as “Rasch delayed” (RD) and “Rasch very delayed” (RVD). Given the difference in both the original Rasch data and the current full set medians compared to the DOD medians, in conjunction with the clear skewing of the data, 4 points was considered adequate to identify delays. Furthermore, the standard errors for the 3-, 4-, and 5-year-olds were .74, .93, and 1.04, respectively. Thus, the delayed evaluations were roughly 4 standard errors below the means for the RD group and 10 standard errors below the means for the RVD group.

### Analysis plan

#### Question 1: Incidence of ELD among a national sample of DOD children ages 3–5 from homes reporting the use of ASL

Frequency distributions (presented above in Table 1) show the distributions of participants being rated for several characteristics. We note that, in the current study, “ratings” constitute the unit of analysis. A number of participants were rated more than once within the 3- to 5-year-old age range, and their multiple ratings are included and treated as separate observations (see Hernandez et al., 2022 for a study of longitudinal change for VCSL participants who were rated more than once). We also note that among the two delay definition groups (Rasch vs. basal difference), two sets of percentages are presented for the age characteristic. Within the Rasch and basal delay groups, percentages show the distributions of each characteristic within the specified delay group as well as within the age cohort. Within the age groups, percentages of ratings indicating a delay, according to the two definitions, are displayed.

#### Question 2: Alternative diagnostic scaling strategies for identifying ELD children

In addition to the simple basal difference determination, two approaches were employed to evaluate differences in the scaling strategies. In the first, we focused on the distributions of Rasch scores (primarily through the use of grouped box plots) for ratings from the different delay groups: (not delayed by either definition; delayed according to the basal score definition only; delayed according to the Rasch definition only; and delayed according to both definitions). We further analyzed the Rasch scores to determine if subgroups of delay categories could be derived. In an additional approach, we focused more deeply on percentages of ratings from each delay group displaying mastery. For this analysis, we utilized the published item sets (Simms et al., 2013) that divide the 114 rating items by the ages at which mastery is expected in the previously indicated item sets 1–5 for native signers. All analyses provide results separately for each age group.

Question 3: Patterns of VCSL ratings indicating those skills that were most often judged to be “NYE” among children identified as having ELD, using the different scaling strategies

Here, we focused more extensively on specific skill items for which a majority of ratings indicated NYE within item sets for ratings of children within different age groups and delay definitions.

The final goal of the study, while not a question, involves developing recommendations for parents and professionals working with children who exhibit ELD based on the data derived

Recommendations based on the data obtained are included in the Discussion section.

## Results

**Figure 1:**
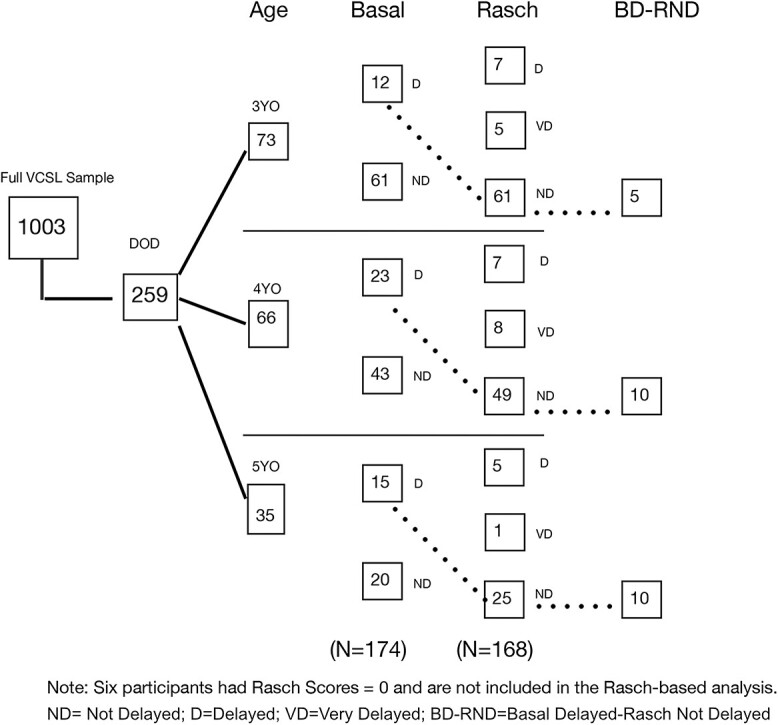
Tabulations of the number of participants in different language delay groups.

This set of analyses focused on ratings of children ages 3–5. As seen in Figure 1, out of the 1,003 unique ratings downloaded for this study, 259 were reported to be from DOD children whose parents reported ASL as the primary language within the home. Of those, a significant number were within the birth to 2-year-old range; 174 were within the 3- to 5-year-old age range. Within this subset, 50 (12 3YO, 23 4YO, 15 5 YO) were determined to have a 2-year delay in ASL skill development based on the basal difference outcomes. Thus, despite having deaf parents with expected early, ongoing ASL modeling, a substantial number of the ratings of DOD children indicated significant language delays according to the basal difference criteria defined above. It should be noted here that these represent individual ratings, not individual children, who may have been rated more than once. Thus, these data are not indicative of the prevalence of ELD in DOD children, but rather that scores reflecting ELD may be observed within a sample of DOD children.

The finding that ratings of a sizeable number of DOD children demonstrated delays of two or more years in their reported basal ASL skills was notable. However, as the published VCSL scoring is limited to the two extreme ratings (mastered and NYE), further analysis of the outcomes of the ratings obtained from the DOD delayed (DOD-D) group were performed using the Rasch scoring, which includes all four points in the ratings of each item. Some ratings that were identified as delayed using the basal difference were not identified as delayed using the Rasch scores. Furthermore, as some children were not able to achieve a basal, a number of ratings did not produce a basal score. A basal difference determination could not be generated for these ratings; however, using the Rasch scoring, these children were identified as delayed. In order for a rating to receive a Rasch score of 0, either multiple items would have missing ratings, or all items would be rated as NYE. This includes items from birth to age 1, for which 80%–100% of the RVD 3-year-old ratings were rated as mastered. Such a score does not appear plausible; thus, these ratings were considered unreliable and removed from further analysis. Of the 174 ratings of 3- to 5-year-olds in the DOD sample, six ratings resulted in Rasch scores of 0 and were eliminated from the Rasch analyses, resulting in a sample of 168 ratings of 101 individual children. Fewer than half (40%) of the ratings were from children rated more than once. Results from children versus ratings are discussed later in the Children versus ratings section below. As seen in Figure 1, a total of 33 ratings, including some not identified as delayed (D) via the basal scores were identified as delayed (RD) or very delayed (RVD) using the Rasch scores (12 3 YO, 15 4 YO, 6 5 YO). Furthermore, 25 ratings (5 3YO, 10 each 4 and 5 YO) were identified as delayed using the basal scores, but were not identified as delayed using Rasch scoring.

Thus, as seen in Table 2, within the cohort of DOD ratings (*n* = 174) identified as reflecting language delays, four groups were identified: those not delayed using any scoring method (ND; *n* = 110), those identified as delayed using the basal difference but not delayed using the Rasch scoring (BD-RND; *n* = 25), those delayed using the Rasch scoring but not basal differences (e.g., those without a basal score; *n* = 14), and those identified as delayed using both methods (*n* = 25). As noted above, of the 39 ratings that were identified as delayed using Rasch scores, six ratings received Rasch scores of zero and were eliminated from further analyses, yielding a total of 33 children identified as delayed using Rasch score. While nearly two-thirds of the ratings of DOD children did not reflect delays using either approach, despite the expectation of ASL exposure from birth a third of the ratings were identified as reflecting delays using at least one approach.

**Table 2:** Language delay groupings for DOD VCSL ratings.

	*N*	%
Not delayed by either definition	110	63.2
Delayed by basal definition only	25	14.4
Delayed by Rasch definition only	14	8.0
Delayed by both definitions	25	14.4
Total	174	100.0

As seen in Table 3, Rasch scores from the original Rasch sample of deaf children (DOH and DOD) ages 3–5 (*n* = 365) demonstrated comparable means and medians for each age group with 3- to about 7-point increases in means per year through age 5. Note that these data include the ratings currently identified as reflecting ELD. On average, in the overall data sets ratings increased by up to an average of 11 points per year for children younger than age 3, resulting in data consistent with a longitudinal study from a subset of the VCSL:O data by Hernandez et al. (2022) from a sample including both DOH and DOD children that would have been expected to include some children with ELD, which reported an average Rasch score gain per year in sequential ratings of children from birth to age 5 as 0.91 points per month. The means and medians for DOD children ages 3–5 were again similar and about 6 points higher than those for the full sample. By definition, the ratings of DOD children with Rasch-identified language delays reflected Rasch scores at least 4 points below those of the overall cohort, resulting in scores approximately 10 points below the DOD cohort.

**Table 3:** Means, medians, and standard deviations of Rasch scale scores for 3-, 4-, and 5-year-olds within different sample segments.

	**3**	**4**	**5**
Original Rasch sample (*N =* 365)			
Mean	56.88	63.89	66.19
Median	57.37	63.23	66.75
Standard deviation	11.24	13.72	11.64
Count	146	136	83
Current full sample (*N =* 584)			
Mean	55.16	62.11	63.55
Median	56.00	61.00	63.00
Standard deviation	11.29	13.17	12.47
Count	235	203	146
Current DOD sample (*N =* 174)			
Mean	62.59	69.48	73.23
Median	63.00	71.00	74.00
Standard deviation	9.51	13.28	10.68
Count	73	66	35

**Figure 2:**
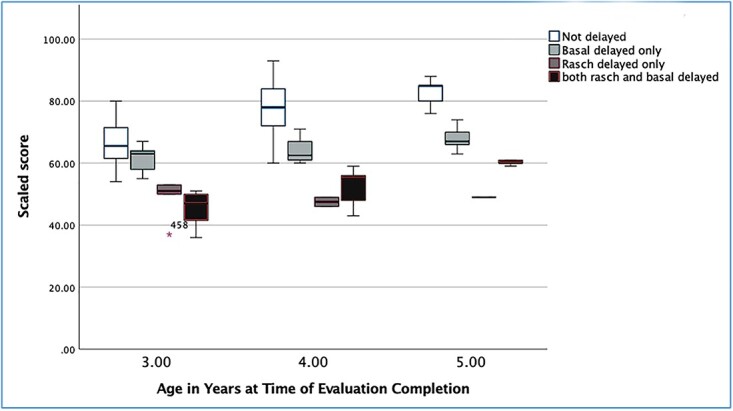
Clustered boxplots of scaled scores by age and delay group.

As can be seen in Figure 2, there is some degree of variance within the cohort of DOD children, the pattern of Rasch scores for the four subgroups: ND (*n =* 110; 56 age 3, 39 age 4, 15 age 5), Rasch delayed only (*n =* 8; 5 age 3, 2 age 4, 1 age 5), basal delayed only (BD-RND, *n =* 25; 5 age 3, 10 age 4, 10 age 5), and both Rasch and basal delayed (*n =* 25; 7 age 3, 13 age 4, 5 age 5). As these ratings are based on the Rasch scores, as seen in Figure 1, the total *N* is 168. While the number of ratings in the Rasch only groups are small, as they both represent Rasch delays, these are combined with the “both Rasch and basal delayed” groups, resulting in somewhat larger total Rasch delay groups (*n =* 33; 12 age 3, 15 age 4, 6 age 5). Those identified as showing no delay were rated as more competent than the other groups, while those only seen as delayed using basal differences (BD-RND) were rated as having somewhat higher skills than those with delayed Rasch scores. By definition, those with delayed Rasch scores (both Rasch only and those rated as delayed using both Rasch and basal differences) were rated as significantly limited relative to the nondelayed and BD-RND cohorts. As these both represented Rasch-determined delays, little can be said about the variability in the outcomes for these groups across the age cohorts. Interestingly, the gap between the nondelayed and basal delayed groups appeared to increase in the older children. While there was significant overlap of the Rasch scores for the ND and BD-RND group for the 3-year-old ratings, this was minimal for the 4-year-old group. Within the 5-year-old cohort, there was essentially no overlap between the ND and BD-RND scores, suggesting that despite being rated as having more age-appropriate language skills than either Rasch delayed group, based on their Rasch scores these children appear to be lagging behind their ND peers.

**Figure 3:**
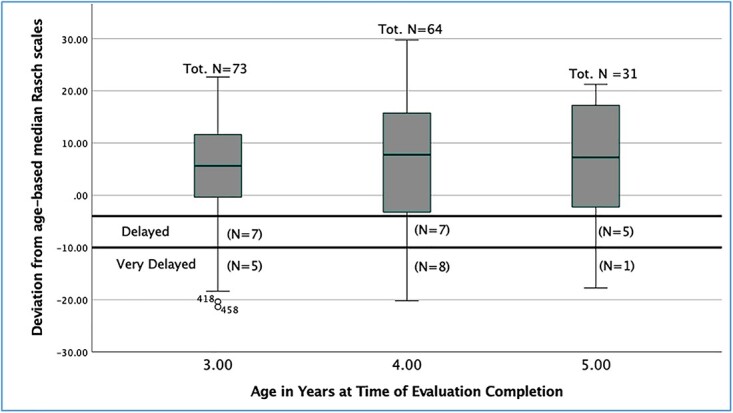
Boxplots of deviations from age-based medians by age in years at time of rating.

Further analyses of the ratings identified as delayed using at least one approach was done using the Rasch scores. In addition to the ND group, as seen in Figure 3, this yielded three subgroups of the DOD cohort: very delayed (RVD; *n =* 14), delayed (RD; *n =* 19), and basal delayed–Rasch not delayed (BD-RND; *n =* 25). Based on their Rasch scores the 19 ratings described as RD were rated as demonstrating skills well below those of their nondelayed peers, while the 14 ratings described as RVD were rated as depicting even more noteworthy limitations in visual language skills.

While the majority of the analyses focused on delays as reflected in ratings of NYE, it is instructive to consider the level of item mastery for the groups of DOD children with and without delays. Table 4 presents the percent of item mastery for the groups identified in Figure 2 for each age cohort. While the ratings indicated that most of the 3-, 4-, and 5-year-olds demonstrated mastery of the majority of the Set 1 (birth to 1-year) items, few of the 3-year-olds in the group identified as delayed using both Rasch and basal scores demonstrated mastery of the Set 2 (1–2-year) items and, regardless of basal score status, none of those identified as delayed using Rasch scores were rated as having mastered any items in Set 5 and a negligible level of mastery was seen for Set 4. Ratings identified as delayed only using basal scores (BD-RND) generally reflected higher levels of mastery than those identified using Rasch scores regardless of whether they were also identified using basal scores. As such high levels of mastery were seen for Set 1 there was little difference in outcomes among the groups; however, for Sets 2–5, ratings identified as ND consistently demonstrated higher levels of mastery than those identified as delayed using either scoring method. Indeed, for the ND group, over 50% item mastery was seen through the item set appropriate for the age (e.g., Set 3 for 3-year-olds). This was not true of the other groups, which didn’t demonstrate over 50% mastery above Set 3 regardless of age.

**Table 4:** Mean percentage of items rated as mastered for each item set by age and delay group.

		**Set 1**	**Set 2**	**Set 3**	**Set 4**	**Set 5**
**Age**	**Delay group**	**Mean**	**Mean**	**Mean**	**Mean**	**Mean**
3	Not delayed (*N =* 56)	99.66	97.83	75.50	38.27	11.47
	Basal delayed only (*N =* 5)	100.00	88.57	40.63	28.57	7.27
	Rasch delayed only (*N =* 5)	94.29	68.57	8.75	0	0
	Both Rasch and basal delayed (*N =* 7)	91.84	36.73	4.46	1.02	0
4	Not delayed (*N =* 39)	100.00	100.00	94.95	71.06	47.47
	Basal delayed only (*N =* 10)	100.00	100.00	58.13	22.86	3.33
	Rasch delayed only (*N =* 2)	40.48	50.00	6.25	0	0
	Both Rasch and basal delayed (*N =* 13)	97.07	63.19	17.55	4.95	0.23
5	Not delayed (*N =* 15)	100.00	100.00	100.00	96.19	69.09
	Basal delayed only (*N =* 10)	100.00	96.43	74.37	37.86	9.39
	Rasch delayed only (*N =* 1)	85.71	50.00	3.13	0	0
	Both Rasch and basal delayed (*N =* 5)	96.24	75.71	33.13	10.00	2.42

Investigation of the Rasch subgroups of delayed (RD) and very delayed (RVD) indicated further differences in mastery outcomes. Although more than half of RVD 4-year-olds were rated as having mastered 9 of the 14 items in Set 2, only two items were mastered by 100% of that group. Few items in Sets 3 or above were mastered by any RVD 4-year-olds. The RD 4-year-olds demonstrated somewhat better item mastery, with most achieving mastery for Set 2 items. Even so, fewer than half of the Set 3 items were rated as mastered for at least 50% of the group and only seven items from Set 4 or above received any ratings of mastered. Only one 5-year-old was rated as RVD, so little can be said about this pattern, but it paralleled that of the RVD 4-year-olds. In general, consistent with their Rasch score–based categorizations, a pattern of minimal item mastery was seen for the RVD 4-year-olds, with greater levels of mastery for the RD group. While the limited item mastery is informative of the upper limits of these children’s skills, the further analyses focused on the ratings of NYE, as this rating indicates an absence of skills that these children should be demonstrating by at least their age-appropriate item set.

Table 5 presents item percentages when 50% or more of ratings were NYE across each age and delay group (ND, BD-RND, RD, and RVD) as a way of directing focus on items that were the most problematic. Items not meeting the 50% criterion were excluded. The rating of NYE was selected to reflect areas where skills were reportedly absent. Thus, these percentages reflect the likelihoods of non-emergence among children at each age and level of delay for each item set. This table also provides the primary category for each item, suggesting the type of functions involved. Within each age group the delay categories are sequenced left to right from RVD (dark gray) to ND (white). Brief descriptions of the content of each item are included. For the full item content, please see Simms et al. (2013).

**Table 5:** 50% percent or greater rated not yet emerging by delay category.

**Set**	**Item**	**Item content**	**Primary**	**3YO (*n =* 5)**	**3YO (*n =* 7)**	**3YO (*n =* 5)**	**3YO (*n =* 56)**	**4YO (*n =* 8)**	**4YO (*n =* 7)**	**4YO (*n =* 10)**	**4YO (*n =* 39)**	**5YO (*n =* 1)**	**5YO (*n =* 5)**	**5YO (*n =* 10)**	**5YO (*n =* 15)**
Ages	#		Category	RVD	RD	BD-RND	ND	RVD	RD	BD-RND	ND	RVD	RD	BD-RND	ND
Set 2	24	Finger babbles	PL	80.0%							none				none
1–2	31	Uses name signs	EL	60.0%				87.5%				X			
	34	Answers WH questions	C	80.0%				50.0%							
Set 3	36	Descriptive classifiers	ASL	100%	85.7%			87.5%							
2–3	37	Nonmanual markers	ASL	60.0%				62.5%							
	38	Points to areas to questions	C	80.0%				50.0%							
	40	Uses pronouns	ASL	100%	57.1%			75.0%							
	41	Names target nouns	EL	60.0%											
	42	Handshapes B, F, O	ASL	80.0%				75.0%							
	43	Uses possessives	ASL	60.0%											
	44	Nonmanual adverbs	ASL	100%	57.1%			100%	71.4%			X			
	45	Names at least three colors	C	60.0%											
	46	Uses >150 signs	EL	100%				75.0%				X			
	47	Lexicalized fingerspelling	ASL	80.0%	57.1%			87.5%	71.4%			X	60.0%		
	48	Three to four sign sentences	EL	100%	100%			87.5%	57.1%			X			
	49	Counts 1–5	C	80.0%											
	50	Uses emotion signs	C	80.0%											
	51	Uses two-step commands	EL	80.0%				62.5%				X			
	53	Uses 250–350 signs	EL	100%	85.7%			87.5%	85.7%			X			
	54	Combines noun & verb	EL	100%				87.5%				X			
	55	Responds to questions	C	100%				62.5%							
	56	Asks two-word question	EL	100%				87.5%				X			
	57	Understands timeline	C	100%	71.4%			75.0%	71.4%			X	60.0%		
	58	Matches colors	C	80.0%											
	59	Uses descriptors	EL	80.0%				62.5%				X			
	60	Enjoys signed stories	C	80.0%											
	61	Multiword sentence	EL	80.0%											
	62	Reads fingerspelling	RL	100%	57.1%			50.0%							
	63	Possessive pronouns	EL	100%				62.5%							
	64	Use basic classifiers	ASL	100%	57.1%			75.0%							
	65	Descriptive classifiers	ASL	100%	71.4%			100%	57.1%						
	66	Tell current stories	C	100%	100%			100%	71.4%			X			
	67	Uses negatives	C	80.0%	71.4%			87.5%				X			
Set 4	68	Verb connects subject & object	EL	100%	85.7%			100%	85.7%	60.0%		X			
3–4	69	Answers questions	C	100%	85.7%			100%	71.4%			X			
	70	Uses verb modification	ASL	100%	85.7%			87.5%	71.4%	50.0%		X	60.0%		
	71	Rhetorical questions	ASL	100%	100%			100%		60.0%		X			
	72	Fingerspells own name	EL	100%	85.7%			87.5%				X			
	73	Uses topicalization	ASL	100%	100%			100%	71.4%			X			
	74	More complex handshapes	ASL	100%	71.4%			75.0%				X			
	75	Part/whole	C	100%	85.7%			87.5%				X			
	76	Understands quantity	C	100%	71.4%			87.5%				X			
	77	Uses 2-of-US; 3-of-US	ASL	100%	85.7%	60.0%		100%	100%			X			
	78	Classifier + action	ASL	100%	85.7%			100%	100%						
	79	Describes needs	EL	100%	85.7%			75.0%				X			
	80	Understands opposites	C	100%	85.7%			87.5%				X			
	81	Complex handshapes	ASL	100%	85.7%			75.0%				X			
Set 5	82	Complex sentences	ASL	100%	100%	80.0%	6.7%	100%	100%	70.0%		X		70.0%	
4–5	83	Counts from 5 to 10	C	100%	85.7%			87.5%	85.7%			X	60.0%		
	84	Holds a conversation	C	100%	85.7%			100%	57.1%			X			
	85	Tells a simple story	C	100%	100%	80.0%	55.4%	100%	100%	80.0%		X	80.0%		
	86	Counts to 15	C	100%	100%	60.0%	71.4%	87.5%	85.7%	50.0%		X	60.0%		
	87	Uses body shift & gaze	ASL	100%	100%	80.0%	55.4%	100%	85.7%	70.0%				60.0%	
	88	Tells stories about life	C	100%	100%	60.0%		100%	100%	60.0%		X			
	89	Answers questions	C	100%	100%			87.5%	100%			X	80.0%		
	90	Understands time	C	100%	85.7%			87.5%	85.7%	50.0%		X			
	91	Sentences with two traits	EL	100%	100%	80.0%	66.1%	100%	100%	60.0%		X	100%	70.0%	
	92	Uses time indicators	ASL	100%	100%			62.5%	71.4%			X			
	93	Spatially arranged storytelling	ASL	100%	100%	100%	71.4%	100%	85.7%	100%		X	80.0%		
	94	Answers what happened/why?	C	100%	85.7%			100%	100%	50.0%		X	80.0%		
	95	Verb modifications.	ASL	100%	100%	100%	62.5%	100%	100%	80.0%		X	100%	60.0%	
	96	Create categories from objects	C	100%	85.7%		51.8%	87.5%	85.7%	60.0%		X	60.0%		
	97	Distinguishes nouns & verbs	ASL	100%	100%	80.0%	69.6%	100%	100%	70.0%		X	80.0%	50.0%	
	98	Understands similarities	C	100%	85.7%	80.0%	62.5%	100%	85.7%	80.0%		X	80.0%	60.0%	
	99	Uses conditionals	ASL	100%	100%	60.0%	62.5%	100%	100%	100%		X	100%	50.0%	
	100	Lists 6+ items to category	C	100%	100%	60.0%	69.6%	87.5%	100%	80.0%		X	100%	60.0%	
	101	Names categories	C	100%	100%	80.0%	71.4%	100%	100%	70.0%		X	100%		
	102	Qualitative descriptors	EL	100%	85.7%		53.6%	87.5%	85.7%	70.0%		X	60.0%		
	103	Tells story in sequence	C	100%	100%		71.4%	100%	100%	90.0%		X	60.0%	60.0%	
	104	Understands parts	C	100%	100%	80.0%	75.0%	100%	100%	60.0%		X	80.0%	70.0%	
	105	Handshape categories	C	100%	85.7%	60.0%	71.4%	100%	100%	80.0%		X	60.0%		
	106	Number distribution	ASL	100%	100%	100%	82.1%	100%	100%	90.0%		X	100%	80.0%	
	107	Sequences items	C	100%	85.7%	80.0%	62.5%	87.5%	100%	60.0%		X			
	108	Identifies outlier object	C	100%	100%	60.0%	67.9%	87.5%	100%	70.0%		X	60.0%	50. %	
	109	Lexicalized signs = handshapes	ASL	100%	100%	100%	76.8%	100%	100%	90.0%		X	80.0%		
	110	Uses WH bracketing	ASL	100%	100%	100%	69.6%	100%	100%	90.0%		X	100%		
	111	Spatial arrangement	ASL	100%	100%	100%	71.4%	100%	100%	80.0%		X	100%	80.0%	
	112	Uses AGENT	ASL	100%	100%	100%	71.4%	100%	100%	80.0%		X	100%		
	113	Use topic continuation	ASL	100%	100%	100%	78.6%	100%	100%	90.0%		X	80.0%		
	114	Understands seasons	C	100%	100%	100%	78.6%	100%	100%	80.0%		X	60.0%		

*Note*. EL = expressive language; RL = receptive language; C = cognitive; ASL = American Sign Language;, SE = social/emotional; PL = prelinguistic; RVD = Rasch very delayed, RD = Rasch delayed; BD-RND = basal delayed–Rasch not delayed; ND = not delayed.

As can be seen in Table 5, only three items within the Set 1 had a majority of children rated as NYE for the RVD 3-year-olds, two items for the RVD 4-year-olds, and one item for the single 5-year-old in the RVD Group. No NYE ratings were observed in item Set 1 or 2 for the RD, BD-RND, or ND groups. Thus, almost all children ages three and older, even those identified as very delayed, would be expected to demonstrate some level of these early language-related skills. Fifty percent or more of the RVD 3-year-olds were rated as NYE for all but two of the 32 items in the Set 3, while this was true of only 12 items for the RD group and no items received this rating for the BD-RND or ND 3-year-olds. All ratings of the RVD 3-year-olds for items in Set 4 and above were NYE. While all items within these sets had majority NYE ratings for the RD group, most were less than 100% for Set 4, suggesting that many of those with milder levels of ELD demonstrate some emergence of skills slightly above their age level. In contrast, only one item in Set 4 had more than 50% NYE ratings for the BD-RND 3-year-old’s ratings and none for the ND ratings for Set 4, reflecting initial emergence of skills in the year above the child’s age. As expected, few of the 3-year-olds, regardless of delay category, demonstrated emergence of skills in Set 5. Thus, as with item mastery, a pattern of diminished proficiency was observed for each of the Rasch-determined delay groups for the 3-year-olds, with the most significant delays being observed in the RVD group and more age-appropriate ratings being seen in the BD-RND group despite their 2-year basal delays. These data contrast with the more limited NYE ratings for the ND ratings.

This pattern was repeated with the 4- and 5-year-old cohorts, with the RVD groups having NYE ratings beginning in Set 2 and most items with 100% NYE ratings starting with Set 3, with all 100% NYE in Sets 4 and 5. While no item in Set 2 had at least 50% NYE ratings, 50% or more of the ratings for the RD 4-year-olds were NYE on nine of the 32 items in Set 3 and all of the items in Set 4 had 50% or more of ratings (but most below 100%) of NYE. The vast majority of the items in the Set 5 were rated as 100% NYE for the RD 4-year-olds. In contrast, only items in Set 4 and above had a 50% or more NYE ratings for the BD-RND group and four items didn’t meet this level within Set 5. No item was rated as NYE for 50% or more of the ND 4-year-olds. Indeed, fewer than 25% of the ratings were NYE on any item even within Set 5 for this group. Only one child in the 5-year cohort was rated as RVD. This child appeared to be quite delayed and presented with as very similar to the RVD 4-year-olds, although several early items in the Set 3 were rated as emerging or mastered. Only three items were rated as 50% or more NYE prior to Set 5 for the RD 5-year-olds, and no item achieved this criterion for the BD-RND group prior to Set 5. Most items in Set 5 received NYE ratings for 50% or more of the RD group, while 13 of the 33 items achieved this level for the BD-RND group. No ratings of NYE were given for any of the ND 5-year-olds.

### Item response patterns

In addition to the pattern of earlier and higher percentages of NYE ratings in the three DOD-D groups, with the most in the RVD group and the least in the BD-RND groups across each age cohort, patterns were noted among the items that were identified as problematic for the groups. Table 6 presents the percentages of items rated as not yet emerging (NYE) within content areas and within VCSL age sets. A total of 168 ratings (of DOD children in ASL homes) of 114 VCSL items, performed on 101 individual participants are reported. The percentages are presented separately by age and by delay status (ND, RD, and RVD). It reveals the systematic *increase* in NYE ratings across the three delay status groups and item sets, and the systematic *decrease* across the age span, from 3 to 5. While some delays were seen in the RD and RVD 3-year-olds, these represented a minority of the ratings in all categories in Set 2. However, starting with Set 3 the vast majority of the RVD 3-year-old ratings reflected limited skills across all three primary categories, suggesting broad ranging deficits. In contrast, for the RD 3-year-olds, as seen in Table 5 more items involving ASL (6) rather than cognitive (3) or expressive (2) and receptive (1) language skills were reported to have 50% or more NYE ratings for this item set despite comparable numbers of ASL (9), cognitive (10), and expressive language (11) items being present when considered by item. When collapsed over the category, as presented in Table 6, nearly half of the ratings within the ASL category were NYE, while a little over a third of the ratings for the other categories were NYE. As can be seen in Table 5, a number of the affected ASL items involve use of classifiers, suggesting that ASL grammar may be a key area of limitation. Multiple items for which this group was rated as 100% NYE, (e.g., using longer sentences and telling stories about current situations), involved both expanded utterances and some form of sentence structure (although not necessarily correct ASL grammar). Additionally, over 85% were rated as having a restricted expressive vocabulary (fewer than 250 signs), suggesting that even the RD 3-year-olds tend to demonstrate more limited expressive language. None of the items in this set were rated as having a majority NYE for the 3-year-old BD-RND group. A similar pattern was seen with the 4-year-old cohort, with items from the same sets, but somewhat fewer items being represented in the RVD and RD groups and the more widespread limitations (nearly 90% of ratings across all three categories being NYE) in the RVD group not observed until Set 4.

**Table 6:** Means of the percentage of items within content groups rated as “not yet emerging” for subgroups defined by age and delay status.

**Total *N =* 168 ratings of 101 participants**	**Age in years**								
	**3**			**4**			**5**		
	**Rasch delay group**			**Rasch delay group**			**Rasch delay group**		
	**ND**	**RD**	**RVD**	**ND**	**RD**	**RVD**	**ND**	**RD**	**RVD**
	**Mean (*N =* 61** ^**48**^ **)**	**Mean (*N =* 7** ^**4**^ **)**	**Mean (*N =* 5** ^**2**^ **)**	**Mean (*N =* 49** ^**21**^ **)**	**Mean (*N =* 7** ^**6**^ **)**	**Mean (*N =* 8** ^**5**^ **)**	**Mean (*N =* 25** ^**13**^ **)**	**Mean (*N =* 5** ^**2**^ **)**	**Mean (*N =* 1** ^**0**^ **)**
Set 1 (birth to 1 year)									
Attention-gaze^9^	.0	.0	.0	.0	.0	1.4	.0	.0	.0
Prelinguistic^4^	.0	.0	.0	.0	.0	.0	.0	.0	.0
Social–emotional^8^	.0	.0	.0	.0	.0	.0	.0	.0	.0
Set 2 (1–2 years)									
Receptive language^3^	.0	.0	20.0	.0	.0	.0	.0	.0	.0
Prelinguistic^3^	.0	4.8	40.0	.0	.0	4.2	.0	.0	.0
Cognitive^4^	.0	3.6	40.0	.0	.0	18.8	.0	.0	.0
Expressive language^4^	.4	14.3	35.0	.0	.0	21.9	.0	.0	25.0
Set 3 (2–3 years)									
ASL^9^	10.0	47.6	88.9	1.8	28.6	75.0	.9	11.1	22.2
Cognitive^11^	5.8	36.4	83.6	1.3	23.4	48.9	.4	12.7	36.4
Expressive language^10^	4.9	34.3	86.0	1.8	20.0	57.5	.8	8.0	60.0
Set 4 (3–4 years)									
ASL^7^	28.8	87.8	100.0	8.5	67.3	91.1	4.6	20.0	85.7
Cognitive^4^	26.6	82.1	100.0	7.1	50.0	90.6	6.0	25.0	100.0
Expressive language^3^	15.3	85.7	100.0	7.5	47.6	87.5	.0	13.3	100.0
Set 5 (4–5 years)									
ASL^13^	68.3	100.0	100.0	27.9	95.6	97.1	20.0	84.6	92.3
Cognitive^18^	58.4	93.7	100.0	21.4	93.7	93.8	14.4	65.6	100.0
Expressive language^2^	59.8	92.9	100.0	18.4	92.9	93.8	18.0	80.0	100.0

*Note*. ND = not delayed; RD = delayed using Rasch estimates of ability; RVD = very delayed using Rasch estimates of ability. Superscripts on the group *N*s indicate the number of individual participants comprising the number of evaluations used in the analysis. The total number of ratings = 168 included the ratings of 101 unique individuals (40% of the total number of ratings analyzed were performed on participants who had duplicate ratings in the dataset). Superscripts on the content areas indicate the number of VCSL rating items comprising that content area. Note that the sum of the superscripts does not equal 114, as content areas within a set containing only one item are not included in this table.

The majority of the RD 3-year-olds were rated as NYE for all but two items in Set 4, understanding opposites (cognitive) and using complex handshapes (ASL). As seen in Table 6, over 80% of the Set 4 ratings within each category were NYE for this cohort. Even so, only two items were rated as 100% NYE, both involving ASL (using rhetorical questions and topicalization). For the 3-year-old ND cohort, 15% (expressive language) to almost 29% (ASL) of the ratings within each category of Set 4 were rated as NYE, with ASL again being the category with the highest percentage of NYE ratings. As seen in Table 5, the BD-RND 3-year-olds had a pattern similar to the ND ratings, but with a higher percentage of NYE ratings. A similar pattern was seen within the 4-year-old ratings, with the RVD group demonstrating widespread limitations starting with Set 4, ND demonstrating minimal NYE ratings across categories until Set 5, and the RD group being in between, again with items involving ASL, particularly ASL grammatical structures, appearing to be present with the greatest percentage of NYE ratings.

### Children versus ratings

Table 6 also depicts the number of children rated as well as the number of individual ratings for this cohort. The number of children (superscript *N*’s) as well as number of ratings are presented for the total and age and delay groups. Note that each child is reported only in the sample for the youngest age of rating. With 40% of the ratings representing children with multiple ratings, this resulted in fewer individual children being represented in the older age groups, including the 0 for the 5-year-old RVD group. This makes the number of older children with Rasch delays appear smaller than the total number of children in each group as some children in these groups had previously been rated. Interestingly, over half of the ND 4-year-olds had previous or additional 4-year-old ratings, while this affected a minority of the RD and RVD children. Regardless, this resulted in a total of 82 (81%) ND children, 12 (11.9%) RD children, and 7 (6.9%) RVD children, or approximately 19% of the individual children being rated as having at least some level of ELD using the Rasch scoring.

## Discussion

DOD children are generally expected to develop ASL skills in a manner paralleling that of hearing children of hearing parents developing spoken language (Lederberg et al., 2013; Lillo-Martin & Henner, 2021). However, even this situation does not ensure the acquisition of language in a timely manner. There are multiple potential sources of language delays and disabilities other than inadequate access to language models or conditions associated with non-hereditary etiologies of deafness, and DOD children would be expected to be as vulnerable as are children in the general population. Thus, our first question was, what is the incidence of ELD/DLD in DOD children? To answer this question, the current study analyzed a sample of 174 ratings of ASL skill development of DOD children ages 3–5 using the VCSL:O. Note that these are individual ratings, rather than individual children, and some children were rated more than once, suggesting that caution should be used in interpreting the incidence of ratings indicative of delays as suggestive of the prevalence of ELD/DLD in DOD children. Even so, while the majority of the ratings (110) indicated expected levels of ASL skills, a sizable minority (64) were determined to reflect significant delays using at least one of the approaches used (basal differences or Rasch scores). Using a basal score delay of 2 years resulted in 50 ratings, or nearly one third of the sample, identified as delayed. While this may reflect an overestimate of delays due to potential issues with the VCSL, such as items that did not fit the overall model of the VCSL or that were out of the sequence based on statistically derived item difficulties (Allen & Morere, 2022), it does not account for the 39 ratings (22%) identified as delayed using the Rasch scores, which remove the effects of such issues. Furthermore, even when the ratings were investigated based on the number of individual children rated rather than the total ratings, 19% of the children were rated as having some level of ELD based on Rasch scores (11.9% RD; 6.9% RVD). This is similar to the number of ELD results for the total number of ratings based on Rasch scores. The finding that 22%–37% of the ratings and 19% of the children were suggestive of significant language delays is concerning, particularly considering the incidence of 11%–18% ELD reported in the general population and only 3%–7% DLD seen in those approaching kindergarten (Chilosi et al., 2021; Laasonen et al., 2018; Sansavini et al., 2021). This raises the question of why a fifth to a third of the ratings in this sample were suggestive of language delays when that is above the expected rate in the general population.

There are a number of possible explanations for these results. First, most of these data are based on individual ratings, not individual children. It is possible that children whose parents or teachers had concerns about their language development were evaluated more frequently than those who seemed to be progressing at the expected rate. However, the fact that over half of the ND 4-year-olds had been rated more than once while less than a third of the combined RD and RVD children were rated previously is inconsistent with this hypothesis. Secondly, the assumption is that deaf parents are fluent in linguistically accurate ASL and incorporate grammatical structures such as reduplication, classifiers, and nonmanual markers in their daily signing; however, as previously discussed, it is possible that a subset of these parents were themselves DOH and either grew up with signed communication, likely both at home and in school, which, while allowing for fluent communication, was not linguistically accurate ASL or that they did not learn to sign until adolescence or later (Singleton & Meier, 2021; Singleton & Newport, 2004; Sümer & Özyürek, 2022). Thus, these parents, while having fluent communication with their deaf children, may not have modeled all the grammatical aspects of ASL that the VCSL:O measures. Note that the norming sample of the VCSL focused on children whose parents were fluent ASL signers. While a sizeable number of the 3- to 5-year-olds in this sample received ratings indicative of ELD, it should be noted that research suggests that despite less accurate early ASL exposure, many deaf children are able to increase the quality of their signing given access to adequate models. Hernandez et al. (2022) found that some, although not all, children whose ASL skills were initially limited began to make rapid gains by age 5, when they entered the K-12 environment. Similarly, Antia et al. (2020) reported that deaf children who initially demonstrated ELD made substantial gains during kindergarten to second grade in their target language (ASL &/or spoken English). Furthermore, as previously noted, DOD children whose parents were late signers eventually surpass their parental ASL models (Singleton & Newport, 2004). Thus, particularly when ELD are related to late or imprecise modeling, the children may “catch up” to their peers when provided with better access to models of linguistically accurate ASL. However, in the studies cited the “catching up” generally occurred upon entrance into kindergarten or later, or at ages 5 and up. As approximately 80% of the ratings in this study involved children ages 3 and 4, a lack of full modeling of ASL grammar and related characteristics could reasonably be seen as a partial contributor to the observed data.

Additionally, it is possible that while many hereditary forms of deafness are not associated with other conditions, some of the children evaluated may have etiologies that are associated with additional conditions that may have increased the incidence of language delays in the cohort. Unfortunately, due to the archival nature of the data, the presence or absence of additional known disabilities was not available.

Another issue related to incidence is the pattern of the ELD findings seen in this sample. Of the Rasch outcomes, only one 5-year-old rating was identified as RVD. This may be partially an effect of the smaller cohort in the 5-year-old group that yielded only six ratings that were indicative of any level of delay using the Rasch scores and only two of these represented children who had not previously been rated. It is also possible that, as noted above, consistent with Hernandez et al. (2022), some of the children who demonstrate delays at younger ages begin to “catch up” when exposed to ASL in the educational setting. Finally, it is possible that some children who demonstrate more significant delays may have been identified with additional conditions affecting their development and been placed in alternative programs due to these conditions as they entered kindergarten. Further investigation of the incidence of language delays in DOD children and the patterns seen in this study is needed.

The second question that was pursued was the use of alternative strategies for identifying language delays in deaf children. As noted above, in addition to the basal differences used in the original VCSL, the authors analyzed the data using standard scores (Rasch scores; Allen & Morere, 2022). Interestingly, 25 ratings identified as delayed by the basal differences were not identified as delayed using this approach, while 14 were identified as delayed using only this approach; 25 were identified using both methods. In addition to avoiding potential effects of poor item sequencing, one of the benefits of the Rasch scoring is that it provides a more nuanced outcome, as it uses all four ratings rather than the two extremes (mastered and NYE). This allows some children who may demonstrate development, but inconsistent use, of many items to achieve higher scores. Those children would reach a ceiling earlier than children who demonstrate mastery, although the overall language skills may be similar. Thus, some children identified as delayed using the basal difference analysis might appear to be within expectations using the Rasch scores. This is consistent with the finding (Table 4) that the BD-RND ratings reflected item mastery more similar to the ND group than those identified as delayed using Rasch scores regardless of their basal status. While future research involving additional measures that could be used to clarify the sensitivity and specificity of the Rasch derived VCSL scores in the identification of ELD/DLD is needed, this suggests that the Rasch scores are likely more accurate than a simple basal difference score.

Similarly, children who did not achieve a basal score could not be evaluated using the basal difference approach, while they could receive a Rasch score. Thus, some children who demonstrated very limited skills, and who would almost certainly be identified as language delayed by those working with the child, would be overlooked by using the strict basal difference approach, but could be identified as delayed using Rasch scores. This is consistent with the observation that in Table 4 those ratings identified as delayed using only Rasch scores generally demonstrated the lowest levels of item mastery of all the groups. In addition to the nuance in identifying those with delays, the Rasch scores subdivided the ratings into those that were very delayed (RVD) as opposed to simply delayed (RD). When reviewing the data, it is quite clear that this group represented a more significantly delayed group, which likely reflects DLD rather than simple ELD. This group likely includes the children who were not able to achieve a basal score due to severely limited item mastery. Such children are more likely to require referrals for additional evaluation rather than simply increased language intervention services. Thus, while the basal difference approach provides a method of identifying language delays among deaf children based on VCSL scores, the Rasch scores appear to provide additional information that can enhance the use of this measure.

A further approach to investigating ELD looked at item mastery in the sample. The percent of each group who demonstrated mastery of individual items was averaged over the full VCSL for each age group across delay categories as demonstrated in Table 4. This analysis indicated that in many ways, while somewhat lower on the more advanced item sets, the BD-RND group was more similar to the ND group than those identified as delayed using Rasch scores regardless of the basal difference status. One clear reflection of this analysis is that the ND outcomes reflected age-appropriate mastery of items within the item sets. This supports the validity of the VCSL as a means of identifying expected development and ELD. While this approach clarifies the differences among the groups identified using the basal difference and Rasch approaches, it may provide little additional information about an individual.

The third question asked related to potential patterns of VCSL ratings indicating those skills that were most often judged to be “NYE” among children identified as having ELD using the different scaling strategies. In addition to investigating the identification of language delays using either the basal difference or Rasch scores, further analysis of the outcomes was pursued looking at the percentages of each group (RVD, RD, BD-RND, and ND) that were rated as NYE for each item. This focus on skills on which the child was not demonstrating even limited performance clearly depicted the range of performance, with the ND ratings indicating that the relevant child was demonstrating at least limited skills in all age-appropriate areas. Those in the BD-RND group had more limitations, but for the most part demonstrated some skills on most age-appropriate items. In contrast, the RVD groups had at least 50% of the protocols rated as at NYE for a majority of items starting with Set 3 even for the 4-year-olds and the sole 5-year-old in this group. The RD group had slightly fewer than half of the items in Set 3 with a majority of NYE ratings for the 3- and 4-year-olds and only two items for the 5-year-olds. This pattern of increasing levels of NYE ratings across the delay groups (RVD > RD > BD-RND > ND) was consistent across all age groups. These data again support the contention that while the basal delay approach provides a reasonable screening tool for identifying deaf children with ELD/DLD, the Rasch scores provide more clarity and nuance that could inform those working with the child about the need for further testing or simply more intensive language intervention.

When investigating patterns at the category and item level, it was noted that ratings identified as RVD demonstrated severe limitations across all item types beginning with age-appropriate items (e.g., Set 3 for 3-year-olds). Thus, they were not only limited in ASL skills, they demonstrated limitations in general language skills and cognitive aspects of communication. Such widespread limitations in communication are suggestive of issues in addition to ELD and are indicative of a need for more in-depth evaluations for these children.

Among the RD cohort a greater percentage of items identified as primarily reflecting ASL rather than cognitive or general receptive or expressive language skills presented with a majority NYE rating. In particular, many of these items involved grammatical aspects of ASL such as use of classifiers, suggesting that while the children may have adequate visual language exposure, it may not consistently involve the full range of ASL characteristics. This is consistent with the finding that although they used similar classifier handshapes, DOD children of late signers used fewer classifiers than DOD children of early signers (Sümer & Özyürek, 2022) This group also demonstrated some delays in expressive language, such as shorter utterances and more limited vocabulary. As the majority of deaf children are born to hearing parents, it is possible that a number of the parents in this DOD group may themselves have been DOH and therefore not had the richness of ASL modeling that those from generationally deaf families may have experienced. They may also not have learned the types of adaptations in early visual language exposure such as tactile cues and short, repetitive utterances that generationally DOD parents use to address the cognitive demands of learning visual language (Lederberg et al., 2013). A similar pattern was seen in the BD-RND group, although with significantly fewer items. These majority NYE ratings seen prior to age-appropriate item sets primarily reflected more advance grammatical structures, such as verb modifications and rhetorical questions. Thus, these cohorts may reflect the effects of less fluent ASL modeling and require increased direct ASL training or modeling rather than broader interventions. This would require monitoring to see if this resulted in significant language gains, but could be an intermediate step prior to further evaluation.

### Recommendations for parents and professionals

As a sizeable portion of the ratings in this sample of DOD children were suggestive of some degree of ELD or even DLD, there is cause for parents and professionals working with these children to monitor the language development of all DHH children, not just those who are DOH. Clearly, these data suggest that the expectation that DOD children are protected from language delays due to expected early, accurate exposure to ASL is unwarranted. While the majority of the ratings in this sample did indeed reflect age-appropriate development, approximately one third did not, and approximately one fifth of the children represented in the sample were identified as delayed using Rasch scores. This raises the question of what parents and professionals should do when the VCSL rating of a child is suggestive of ELD. Certainly, the VCSL is only one piece of data and should be interpreted in the context of other information about the child. For example, is the child’s etiology suggestive of the potential presence of additional conditions? Or are the parents themselves DOH? In the former case, further evaluations may be warranted, while in the latter case, the linguistic accuracy of the parents might be investigated.

With the additional approaches to analysis proposed in this paper, there may be further clues to how parents and professionals may wish to proceed. While the standard basal deficit approach may identify issues, the Rasch scoring can help to determine if this reflects a severe delay, suggestive of a DLD and possible other issues that should be investigated, or a milder ELD that may be ameliorated by more intensive ASL intervention. A child whose Rasch score is RVD would likely best be served by referral for a multidisciplinary assessment to determine what factors are producing the widespread, severe delays observed in the rating. In contrast, a child whose Rasch score is suggestive of milder delays, particularly younger children, may be appropriate for a review of potential environmental factors, such as the quantity and linguistic quality of the language modeling combined with more intensive ASL modeling and instruction in the educational setting. Research has suggested that children who initially obtain lower scores on early measures of ASL may “catch up” when provided with appropriate ASL models (Hernandez et al., 2022). Indeed, Singleton and Newport (2004) studied a DOD child whose parents were late signers and whose signing was at a non-native level. They found that the child exposed to early, but not linguistically accurate, ASL was able to develop more native-like ASL skills by age 7. These children should be regularly rated with the VCSL or a similar instrument in order to determine if the interventions provided are effective or if further evaluations may be needed.

An additional approach to considering the child’s needs is item analysis of the rating, particularly looking at both the items on which they demonstrated mastery and those rated as NYE. A child who demonstrates mastery of more cognitive and general language skills in the presence of a large number of NYE ratings for ASL skills may again be a candidate for more intensive ASL instruction and modeling, while a child who demonstrates more widespread NYE ratings may need additional testing to determine if there are cognitive, behavioral, environmental, or physical issues affecting their language development. For a DHH child learning ASL, there are not only the potential sources of ELD and DLD such as biologically based language disorders, ASD, and ID. Factors such as vision or visual processing issues can also hinder ASL development. Similarly, attention issues, such as ADHD, while unlikely to produce severe language delays, can limit a child’s ability to take advantage of the language modeling available, producing some degree of ELD. Thus, while a child whose VCSL Rasch score is RVD suggests the need for an evaluation referral, those with milder suggested delays will likely benefit from consideration of factors such as vision and attention and the quality and quantity of ASL modeling in conjunction with ongoing monitoring and more intensive language intervention at school.

### Limitations and future research

There are a number of limitations to this study. First, the ratings used in this study represented a subset of an anonymous database of ratings performed for educational and early intervention purposes for which the parents agreed to research use. As this was an archival data set, only the information collected when the ratings were performed was available for analysis. While the raters were reported to be primarily teachers of the deaf, early interventionists, and related professionals, specific information related to individual raters, including job title and hearing status, was not available. Furthermore, although the VCSL was designed to be administered by trained raters and later access was restricted to those with formal VCSL training, the specific training of the raters for this cohort was not available. Additional information, such as whether the parents themselves were DOD, parental ASL skills and age of exposure to ASL, and other data would have been beneficial to the interpretation of the observed outcomes. For example, no information which could have provided converging evidence of the children’s language/ASL skills, such as data from other assessments or independent assessments of the participants was available. Such data would have enabled more definitive interpretation of the current data. Future research in which broader demographic information, as well as more detailed information about rater training and characteristics (e.g., job title and hearing status), was available would add significantly to the types of recommendations that could be provided based on the outcomes. Additional measures, such as evaluation of parental ASL skills, videos of parent–child communication in the home, and additional language/ASL assessments of the children would be beneficial, as would information about early intervention and programming for the children to determine if differences emerge between the ND and the ELD groups.

For most of the analyses, the ratings used represented individual ratings, not individual children. Some of the children were evaluated more than once, with 40% of the ratings representing such children. It is possible that the incidence of ELD in this sample of DOD children was overestimated due to children for which there were concerns being rated more frequently than those who appeared to demonstrate more typical ASL development. However, even when the data were examined based on the number of children rated rather than total ratings, a sizeable portion (19%) appeared to demonstrate some degree of ELD. Regardless, further investigation of the incidence of ELD in DOD children is needed to determine if the current data are reflective of a greater risk relative to the general population or if this is secondary to the data set obtained. Furthermore, while the sample itself is relatively large for research with DOD children, as the children are subdivided by age and delay category, some of the sample sizes are quite small. For example, only five 3-year-old and one 5-year-old ratings were identified as RVD. While this represents a positive in that this category represents serious delays, this means that caution should be used in interpreting the data. Research using data collected specifically for the study, for which the additional demographics and measures such as additional evaluations of child language/ASL skills and parental sign skills would allow for converging evidence to clarify the accuracy, sensitivity, and specificity of the Rasch scores and clarification of the sources and types of language delays seen in DOD signing children,

Additional areas of future research include further investigation of the differences between children whose ratings fall into the RVD versus RD categories. Questions could include the following: (1) Do the RVD children tend to remain significantly delayed, or do they tend to improve with intervention? (2) Are the RVD children more likely to be identified as having a DLD or other developmental disabilities? (3) Are there differences in the trajectories of ASL development between the RVD and RD groups? In addition to research focusing on DOD children, research similar to the current study should be pursued with DOH children. These children may be more likely to experience ELD due to limitations in ASL modeling and may also be more vulnerable to DLD or other developmental disabilities due to etiologies that may have associated co-occurring conditions known to be less prevalent among DOD children (Crump & Hamerdinger, 2017). Similarities and differences in the incidence and patterns of ELD in DOD and DOH children could aid in our understanding of both the dynamics of early language development and the monitoring and interventions required for both populations.

In conclusion, the expectation that DOD children will have typically developing skills is problematic, as it may result in delays in monitoring, identification, and intervention for early language in these children. While deaf children, regardless of parental hearing status, may vary in the trajectories of their early language development (Hernandez et al., 2022), even those whose apparent ELD may represent normal variance in developmental trajectories should be monitored to ensure that valid delays needing intervention are not overlooked. Although the VCSL represents only one measure of language development in deaf, signing children, the current data, as well as previous research indicating DLD in DOD children (e.g., Mason et al., 2010; Morere, 2003; Quinto-Pozos et al., 2017) are suggestive of potential issues in sign language development in DOD children. These findings suggest that while many DOD children have typically developing ASL skills, a sizeable percentage of these children may present with ELD or even DLD. Thus, all young deaf children’s language development should be monitored in order to offer timely intervention or investigation of potential additional conditions that may affect language development.
